# Comparison of analyses of the QTLMAS XII common dataset. II: genome-wide association and fine mapping

**DOI:** 10.1186/1753-6561-3-s1-s2

**Published:** 2009-02-23

**Authors:** Lucy Crooks, Goutam Sahana, Dirk-Jan de Koning, Mogens Sandø Lund, Örjan Carlborg

**Affiliations:** 1 Department of Animal Breeding and Genetics, Swedish University of Agricultural Sciences, Box 7023, SE-75007 Uppsala, Sweden; 2 University of Aarhus, Faculty of Agricultural Sciences, Department of Genetics & Biotechnology, Research Centre Foulum, DK-8830, Box 50, Tjele, Denmark; 3 The Roslin Institute and R(D)SVS, University of Edinburgh, Roslin Biocentre, Roslin, Midlothian, EH25 9PS, UK

## Abstract

As part of the QTLMAS XII workshop, a simulated dataset was distributed and participants were invited to submit analyses of the data based on genome-wide association, fine mapping and genomic selection. We have evaluated the findings from the groups that reported fine mapping and genome-wide association (GWA) efforts to map quantitative trait loci (QTL). Generally the power to detect QTL was high and the Type 1 error was low. Estimates of QTL locations were generally very accurate. Some methods were much better than others at estimating QTL effects, and with some the accuracy depended on simulated effect size or minor allele frequency. There were also indications of bias in the effect estimates. No epistasis was simulated, but the two studies that included searches for epistasis reported several interacting loci, indicating a problem with controlling the Type I error rate in these analyses. Although this study is based on a single dataset, it indicates that there is a need to improve fine mapping and GWA methods with respect to estimation of genetic effects, appropriate choice of significance thresholds and analysis of epistasis.

## Background

For decades, geneticists have used genetic linkage to identify and locate genomic loci that determine traits with Mendelian as well as complex genetic inheritance. The most common approach to genome-wide genetic analyses has been to utilize the extended linkage disequilibrium that exists in pedigrees to screen the genome using a few hundred markers or even less. Such linkage analysis (LA) studies are powerful and in experimental pedigrees they allow detection of loci of moderate effect and with complex inheritance patterns, including imprinting and epistasis [1]. Using this approach, large numbers of loci have been mapped [2]. LA uses only the recombination events that have occurred within the pedigree of genotyped individuals and this limits the resolution to regions tens of megabases long, containing potentially hundreds of genes. New molecular methods now allow cost-efficient genotyping of several hundred thousand genetic markers, making it possible to increase resolution by using the short-range linkage disequilibrium (LD) in general populations regardless of the pedigree structure. This LD mapping approach has been coined genome-wide association (GWA) when applied to whole-genome scans and has revolutionized the field of complex trait genetics in human populations [3]. It also opens up new opportunities for high resolution mapping in animal populations [4]. Pedigree-based association methods that attempt to take into account the general relationships between related individuals have also been developed. GWA is considered to be more powerful than LA for detecting the effects of common alleles with small effects but is less powerful when traits have a complex genetic determination, including epistasis. A relatively recent proposal is to combine elements of both LA and LD mapping into one analysis, known as LDLA (combined linkage disequilibrium and linkage analysis) [5]. An alternative to classical statistical approaches is a Bayesian analysis, which has the advantage that determining the number of QTL that should be modelled can form an integral part of the process and effects of all QTL can be estimated simultaneously [6].

The purpose of distributing a common dataset to participants of the QTLMAS XII workshop was to evaluate current and new methods for QTL analysis and genomic selection by their performance on the same data. The distributed dataset included dense marker genotypes of individuals in a deep pedigree. Here, we summarise and assess the six studies that focused on QTL mapping [7-12]. Our aim was to identify the strengths and weaknesses of the different methods and try to draw more general conclusions about the types of approach that perform best, as well as highlighting areas that need more research. The results from the studies relating to genomic selection are evaluated in the second summary paper of this supplement [13].

### The dataset

**Figure 1:**
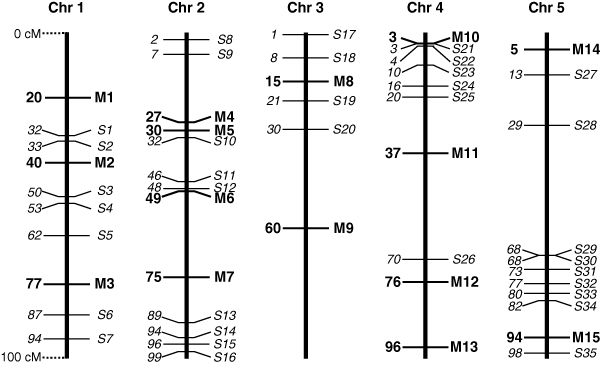
**Chromosomal positions of simulated QTL**. Each simulated chromosome (Chr) is 100 cM long. QTL are indicated on the right-hand side of each chromosome and their position in cM on the left-hand side. No QTL were simulated on chromosome 6.

The data available for fine-mapping and genome-wide association analyses consisted of a simulated four-generation pedigree of 4,665 individuals [13]. Phased biallelic marker genotypes were given at 0.1 cM intervals for six chromosomes, each 100 cM long. Hence, chromosome-wide haplotypes containing 1,000 markers per chromosome were given. Fifty biallelic QTL with additive effects were simulated. Details of these QTL are given in Table 1 and their genomic locations are illustrated in Figure 1. For six QTL, the location was pre-defined and their effects were chosen so that the QTL explained a fixed proportion of the genetic variance. The genetic variance for each QTL was calculated as 2***p***(1 - ***p***)***α***^2^, where ***p ***is the minor allele frequency in the four generations and ***α ***is the average effect of allelic substitution (average change in genotypic value when one allele is randomly substituted for the other, which we calculated from the data using the formula in [14]). The locations and effects of the remaining QTLs were randomly sampled. A normally distributed error term was added to the genetic value for each individual to give a genetic variance of 0.3 times the phenotypic variance. No QTL were simulated on chromosome 6, making it a control for false positives. The number of QTL simulated on chromosomes 1–5 was 10, 13, 6, 10 and 11, respectively. None of the QTL was located at a marker position and therefore the QTL genotypes were unknown to the participants. The average effect of allelic substitution for the QTL varied from <0.01 to 0.75. One QTL was, by chance, fixed in the base population; the minor allele frequencies of the other QTLs ranged from 0.04 to 0.47.

**Table 1:** Simulated QTL.

**Name^a^**	**Chr^b^**	**Location (cM)**	**Effect^c^**	**Minor allele frequency**	**Genetic variance**	**% of phenotypic variance explained**	**Estimated effect in multiple regression**
M1	1	20.00	0.62	0.28	0.15	3.50	0.61
S1	1	31.87	0.01	0.44	0.00	0.00	0.06
S2	1	33.16	0.00	0.30	0.00	0.00	0.04
M2	1	40.00	0.56	0.07	0.04	0.91	0.62
S3	1	50.37	0.06	0.46	0.00	0.04	0.08
S4	1	52.50	0.05	0.40	0.00	0.03	0.07
S5	1	62.21	0.00	0.29	0.00	0.00	0.02
M3	1	77.23	0.37	0.29	0.06	1.29	0.42
S6	1	86.68	0.01	0.30	0.00	0.00	0.09
S7	1	93.99	0.01	0.47	0.00	0.00	0.01
S8	2	2.25	0.01	0.39	0.00	0.00	0.06
S9	2	6.52	0.07	0.38	0.00	0.06	0.09
M4	2	27.41	0.35	0.44	0.06	1.38	0.44
M5	2	30.00	0.33	0.21	0.04	0.82	0.25
S10	2	32.49	0.04	0.41	0.00	0.02	0.07
S11	2	45.71	0.01	0.09	0.00	0.00	0.07
S12	2	48.22	0.04	0.08	0.00	0.01	0.06
M6	2	48.62	0.37	0.40	0.07	1.50	0.39
M7	2	74.91	0.50	0.18	0.07	1.63	0.46
S13	2	89.04	0.12	0.22	0.01	0.12	0.15
S14	2	93.54	0.25	0.32	0.03	0.61	0.22
S15	2	95.66	0.02	0.29	0.00	0.01	0.12
S16	2	97.83	0.13	0.41	0.01	0.19	0.14
S17	3	0.70	0.03	0.00	0.00	0.00	-^d^
S18	3	7.89	0.01	0.46	0.00	0.00	0.04
M8	3	14.91	0.30	0.40	0.04	0.98	0.27
S19	3	21.07	0.02	0.26	0.00	0.00	0.00
S20	3	29.81	0.07	0.29	0.00	0.04	0.05
M9	3	60.00	0.68	0.07	0.06	1.29	0.70
M10	4	3.21	0.61	0.39	0.18	4.01	0.64
S21	4	3.44	0.08	0.32	0.00	0.06	0.10
S22	4	3.88	0.02	0.23	0.00	0.00	0.02
S23	4	10.00	0.01	0.04	0.00	0.00	0.06
S24	4	16.35	0.00	0.36	0.00	0.00	0.11
S25	4	19.84	0.07	0.47	0.00	0.05	0.10
M11	4	36.93	0.34	0.24	0.04	0.95	0.37
S26	4	69.56	0.00	0.08	0.00	0.00	0.01
M12	4	76.06	0.58	0.41	0.16	3.70	0.58
M13	4	96.49	0.29	0.19	0.03	0.59	0.38
M14	5	5.15	0.18	0.21	0.01	0.24	0.25
S27	5	12.98	0.09	0.44	0.00	0.10	0.09
S28	5	28.64	0.00	0.13	0.00	0.00	0.05
S29	5	68.39	0.12	0.44	0.01	0.15	0.17
S30	5	68.48	0.00	0.43	0.00	0.00	0.02
S31	5	72.54	0.00	0.12	0.00	0.00	0.06
S32	5	77.02	0.13	0.25	0.01	0.14	0.15
S33	5	80.00	0.08	0.11	0.00	0.03	0.05
S34	5	82.14	0.01	0.36	0.00	0.00	0.08
M15	5	93.50	0.75	0.26	0.22	4.97	0.75
S35	5	98.32	0.01	0.45	0.00	0.00	0.02

^a^QTL labelled M are major QTL, known to be detectable in this dataset based on the results from our multiple regression. QTL labelled S are secondary QTL that were not detected, with the significance threshold used, in our multiple regression. ^b^Chromosome. ^c^Average effect of allelic substitution (absolute value). ^d^Could not be estimated because the QTL was fixed in the population.

### Description of studies and treatment of results

Descriptions of the methods used to produce the results that we compared are given in Table 2, along with the notation that we will use to refer to each study. All of the studies described more than one analysis. Mostly, one analysis was clearly preferred or a final set of results was given and these were used in our comparison. Two studies did not give a clear preference for one method and we chose results based on their comments. From LDLA1, we took the positions and effects from the multiple regression that were located in regions found by LDLA and 23.2 cM on chromosome 1, which was found only by LDLA. Two methods were used in LDHap: a haplotype method called Blossoc and single marker association. Each was applied to both the raw data and the data pre-corrected for pedigree, sex and generation. The results from Blossoc did not include estimates of QTL effects. For our comparison, we took the positions identified by Blossoc with pre-corrected data and 13.3 cM on chromosome 3, which was close to the chosen significance threshold for Blossoc with pre-corrected data, detected by Blossoc with raw data and by single marker association with both raw and pre-corrected data. For the estimates of QTL effects, we took the values from single marker association with pre-corrected data that corresponded to these positions.

**Table 2:** Summary of studies.

**Study**	**Type**	**Loci**	**Type of loci^a^**	**Loci effects**	**Additional effects**	**Model**	**Comments**
LABayes [7]	LA	multiple	multi-marker^b^	fixed, additive	sex + generation	Bayesian	only every tenth marker used

LDBayes [8]	LD	multiple	single marker	random, additive	-	Bayesian	

LDLA1 [9]	LD	multiple	single marker	fixed, additive +dominance	polygenic	mixed model, REML^c ^and FS^d^	markers selected by allele frequency difference in high/low offspring per sire
	LDLA	single	haplotype (2)	random, additive	polygenic	variance component, REML	markers selected by allele frequency difference in high/low offspring per sire

LDmulti [10]	LD	multiple	single marker	fixed, additive +dominance +epistasis	pre-correction for polygenic + sex + generation	linear regression, least squares and FS	mixed model, REML used for pre-correction

LDHap [11]	LD	single	haplotype (10^e^)	fixed, additive	pre-correction for polygenic + sex + generation	phylogeny building, cluster analysis^f^	mixed model, ML^g ^for pre-correction, maximum in 10 cM interval
	LD	single	single marker	fixed, additive	polygenic + sex + generation	mixed model, ML	maximum in 5 cM interval, explored epistasis

LDLA2 [12]	LA	two	single marker	random, additive	polygenic	variance component, REML	only marker data from last two generations used
	LDLA	single	haplotype (10)	random, additive	polygenic	variance component, REML	only marker data from last two generations used, only on most significant region per chromosome from LA
	LD	single	haplotype (3)	random, additive	polygenic	variance component, REML	only marker data from last two generations used, only on most significant region per chromosome from LA

^a^In brackets is the number of markers in a haplotype. ^b^Probability of QTL genotype is conditional on all marker data (at reduced 1 cM density) for the chromosome. ^c^Restricted maximum likelihood. ^d^Forward selection. ^e^At least 10 markers used. ^f^Using Blossoc [16]. ^g^Maximum likelihood.

Three of the studies aimed to identify and estimate the genetic effects of as many simulated QTL as possible, whilst controlling the Type I (false positive) error rate. The methods used included Bayesian linkage analysis (LABayes), multiple regression LD analysis (LDmulti) and an LD approach that scores the clustering of phenotype with reconstructed phylogeny (LDHap). The remaining three studies had slightly different goals. Two aimed to find the most important QTL efficiently with a reduced analytic effort. A selective genotyping strategy was explored in LDLA1, choosing which markers to include based on allele frequency differences between individuals with high and low phenotypic values. A limit of two QTL per chromosome was applied in LDLA2 and only marker data from the last two generations of the pedigree was used. LDLA1 used LDLA in combination with multiple regression LD analysis and LDLA2 applied LDLA and LD analysis only to the most significant region per chromosome identified by linkage analysis. The last study (LDBayes) came from a genomic selection perspective, directed at finding all potential QTL, and did not aim to control the type 1 error rate. In order to make a more equivalent comparison with the other studies, we only included the positions from LDBayes with the largest estimated effects (see below for more details). Genetic effects in LDBayes were estimated with a Bayesian LD analysis.

## Methods

### Assessment of QTL detectability

Because the dataset is the result of a single simulation, detection of QTL may be limited by the minor allele frequencies generated in that simulation. Detectability will also depend on the size of the QTL effect and be affected by the population size. Therefore, to determine which QTL are potentially detectable in this dataset, we fit a multiple regression with all 50 QTL genotypes. Our rationale was that QTL that could not be identified when the correct genetic model was used, could not be correctly identified by the participants. The purpose of fitting a multiple regression was to exclude spurious effects due to linkage with another QTL of large effect, which might have been found in single locus analyses. We used the following linear model of purely additive effects.

***y ***= *μ *+ ***Xα ***+ ***e***,

where ***y ***is the vector of phenotypes, μ is the population mean, ***X ***is a 50-column matrix of indicator variables for the genotype of each QTL, ***α ***is a vector of average effects of allelic substitution for the QTL and ***e ***is a vector of normally distributed error terms. The indicator variables were set to -1, 0 and 1 for QTL genotypes 11, 12 and 22, respectively. We declared a QTL significant if the *p*-value for a *t*-test of the estimated effect was <8 × 10^-6^, which is a Bonferroni correction for an overall significance level of 0.05 with 6,000 tests. Although the Bonferroni correction is conservative, the next smallest *p*-value was 3 × 10^-4^, so only a much more lenient threshold would have resulted in more QTL found, and the relevant QTL was not reported by any of the studies. The significance thresholds used in each of the studies are given in Table 3. To gain an understanding of which QTL were detected by each study, we also carried out individual regressions on QTL genotype.

**Table 3:** Threshold criteria used in the studies and description of epistatic analyses.

**Study**	**Threshold** **criteria**	***P*-value**	**Comments**
LABayes	2× ln(Bayes factor) ≥ 3	0.08	We equated 2× ln(Bayes factor) with a likelihood ratio test statistic with one degree of freedom [17] and calculated the *p*-value with a χ^2 ^approximation. This method compared models with an increasing number of QTL, for each chromosome, therefore far fewer tests were necessary than for the other studies.

LDBayes	-	-	No significance tests were performed.

LDLA1	LD: F > 4^a^	0.007	Tests were only performed on markers that had a significant difference in allele frequency between high/low offspring from each sire at p < 0.0016.
	LDLA: LRT^b ^> 12.8	0.0003^c^	Tests were only performed on markers that had a significant difference in allele frequency between high/low offspring from each sire at p < 0.0016.

LDmulti		8×10^-6^	An epistatic analysis was performed. An epistatic model was tested against two-locus marginal model (*p *< 8 × 10^-6^) for pairs of markers significant alone, against one locus model (*p *< 1 × 10^-7^) for one significant and one non-significant marker, and null model for pairs of non-significant markers (*p *< 3 × 10^-9^). In last two cases, epistatic model was then tested against two-locus marginal model at *p *< 2 × 10^-5 ^and *p *< 5 × 10^-7^, respectively.

LDHap	haplotype: HQ^d ^ ≥ 15	2×10^-9e^	
	single marker: LRT >32.8	10^-8^	An epistatic analysis was performed using single marker association on pre-corrected data. For each 1 cM interval the marker in highest average LD with the others in the same interval was found. Each type of epistatic interaction (e.g. additive by dominance) was then tested for pairs of these markers at *p *< 10^-3^. When these were significant epistasis was tested for all pairs of markers in the two intervals at *p *< 10^-6 ^and pairs within 10 cM of each other were excluded.

LDLA2	LRT > 6	0.014^c^	-

^a^Lowest F-to-enter value reported. ^b^Likelihood ratio test statistic. ^c^From a χ^2 ^approximation with one degree of freedom. ^d^Hannan-Quinn criteria, which is similar to 2× ln(Bayes factor). ^e^From regression of *p*-values obtained by permutation test (10^8 ^replicates) for chromosome 1 with raw data against HQ score (Ledur, pers. comm.).

### Criteria for detection of a simulated QTL

We will refer to the QTL that were detected by our multiple regression model as M-QTL (for major QTL) and the remaining QTL as S-QTL (for secondary QTL). Participants were considered to have correctly identified a QTL if they reported a position within 5 cM either side of an M-QTL. If a reported QTL was within 5 cM of more than one M-QTL, we treated this as detection of the M-QTL that was closest. If more than one QTL was reported within 5 cM of an M-QTL, we took the closest reported QTL as the estimate of the M-QTL and treated the others as false positives. Other reported QTL, including those close to S-QTL, with two exceptions described in the results, were treated as false positives. To select which positions should be included from LDBayes, we first ranked them by estimated effect size. We then chose a cut-off such that 10 M-QTL were detected.

### Measures for comparing the reported results

The results of the participants were compared in several ways. First we determined how many M-QTL were detected and the number of false positives. Second, we looked at accuracy in estimates of QTL position, by calculating the absolute difference between the reported and simulated position. For LABayes we took the mode of each identified region as the estimate of QTL position. Third, we considered accuracy in estimates of effect size. We determined the magnitude of the difference between the estimated and simulated effect as a percentage of the simulated effect. Most of the studies reported an estimate of the additive effect. For LDLA1 and LDmulti, we took half the difference in genotypic value between the 11 and 22 genotypes as the estimate of effect size. For the simulated effect, we used the average effect of allelic substitution calculated from the data. As an equivalent measure of error for LDLA2, we calculated the magnitude of $100\times \left(\sqrt{V_{est}/V_{sim}}-1\right)$, where V_est _is the estimated genetic variance explained by the QTL and V_sim _is the simulated genetic variance. We also looked at tendencies for bias in reported effects. For QTL detection, position and effect accuracy, we evaluated potential relationships with simulated QTL effect, percentage of phenotypic variance explained by the QTL and minor allele frequency (MAF).

## Results

### Power of QTL detection

15 of the 50 simulated QTL were significant in our multiple regression on known genotypes and therefore were potentially detectable using a stringent genome-wide significance threshold (Table 4). These were the QTL with the 14 largest simulated effects and the 16^th ^largest simulated effect. All except the last were also significant in individual regressions.

**Table 4:** Comparison of M-QTL and reported QTL.

						**Estimated QTL**												
**Simulated QTL**																		
						**MR^f^**	**LABayes^g^**		**LDBayes^i^**		**LDLA1^j^**		**LDMulti**		**LDHap**		**LDLA2**	
**QTL**	**Chr^a^**	**Loc^b^**	**e^c^**	**p^d^**	**Vg^e^**	**e**	**Loc**	**e^h^**	**Loc**	**e^h^**	**Loc**	**e^k^**	**Loc**	**e^k^**	**Loc**	**e^h^**	**Loc**	**Vg**

M1	1	20.0	0.62	0.28	11.8	0.61	21	0.55	19.5	0.66	23.2	-^l^	19.6	0.31	20.0	0.71	19.5	0.12
M2	1	40.0	0.56	0.07	3.1	0.62	41	0.67	39.3	0.59	41.5	0.41	40.2	0.12	40.2	0.78		
M3	1	77.2	0.37	0.29	4.4	0.42	76	0.30	77.7	0.48			77.8	0.23	77.8	0.40	76.6	0.04
M4	2	27.4	0.35	0.44	4.7	0.44			24.9	0.43			27.0	0.22	26.7	0.43	26.0	0.12
M5	2	30.0	0.33	0.21	2.8	0.25	29	0.58			32.6	0.22						
M6	2	48.6	0.37	0.40	5.1	0.39	50	0.46	48.2	0.42	48.3	0.29	48.3	0.18	48.7	0.45	53.2	0.10
M7	2	74.9	0.50	0.18	5.5	0.46												
M8	3	14.9	0.30	0.40	3.3	0.27							13.3	0.16	13.3	0.35	11.9	0.07
M9	3	60.0	0.68	0.07	4.4	0.70									60.1	-^l^		
M10	4	3.2	0.61	0.39	13.6	0.64	4	0.78	3.4	0.55	3.3	0.49	3.3	0.33	3.2	0.59	3.1	0.49
M11	4	36.9	0.34	0.24	3.2	0.37			36.3	0.40								
M12	4	76.1	0.58	0.41	12.5	0.57	77	0.50	76.5	0.52	76.5	0.50	76.5	0.24	76.5	0.55		
M13	4	96.5	0.29	0.19	2.0	0.39	98	0.41	99.2	0.4	96.5	0.32			95.2	-^l^		
M14	5	5.1	0.18	0.21	0.8	0.25	2	0.35										
M15	5	93.5	0.75	0.26	16.8	0.75	95	0.72	95.5	0.6			93.5	0.36	93.5	0.63	93.9	0.18

^a^Chromosome. ^b^Location. ^c^Average effect of allelic substitution (absolute value). ^d^Minor allele frequency. ^e^Percentage of genetic variance explained by the QTL. ^f^Multiple regression on known QTL genotypes. ^g^One QTL was falsely identified at 10 cM on chromosome 4. ^h^Reported estimate of the additive effect. ^i^Only the positions with the 16 largest effect estimates were included. Five QTL were falsely identified at 52.6 cM on chromosome 1, 65.4 cM on chromosome 3, and 3.0, 3.7 and 75.8 cM on chromosome 4. ^j^Two QTL were falsely identified at 3.1 and 4.8 cM on chromosome 4. ^k^Half the difference between the estimated genotypic value of the 22 and 11 genotypes. ^l^Not estimated.

Eleven M-QTL were found in LDHap (Table 4). Ten M-QTL were found in LABayes and by design, in LDBayes, and nine were reported in LDmulti. The studies with the lowest power were LDLA1 and LDLA2, the two with restricted analyses, in both of which only seven QTL were identified. Furthermore, for one of the cases in LDLA2, the M-QTL (M6) was outside the confidence interval for the estimated position.

### Type I error rate

There were no false positives reported in LDLA2, or in the marginal effects reported in LDmulti and LDHap. One false positive was found in LABayes (Table 4). A second region reported in this study (91–99 cM on chromosome 2) was not treated as a false positive because it contained three minor QTL, whose combined effect was significant in our multiple regression model. Two false positives were given in LDLA1. For the cut-off that we applied, there were five false positives for LDBayes. LDBayes also reported a QTL at 94.9 cM on chromosome 2, which, for consistency with our treatment of LABayes, we did not consider a false positive.

### Key features of discovered QTL

**Figure 2:**
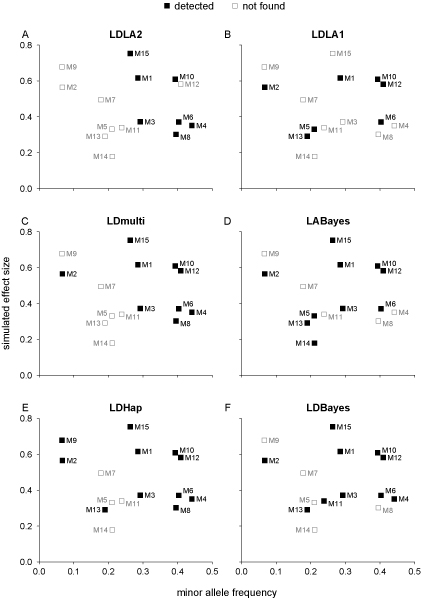
**QTL detected by each study**. In (A)-(F) the M-QTL identified, and not found, by each study are shown, in relation to the simulated average effect of allelic substitution and minor allele frequency.

We looked for trends in which QTL were detected. Figure 2 shows the M-QTL, ordered by simulated effect size and minor allele frequency that were found by each study. The QTL found in LDLA2 were those with highest minor allele frequency (MAF) for a given chromosome in the last two generations. In LDmulti and LDHap, the same QTL plus two more (M2, M12) were detected. M2 and M12 had large simulated effects and were the next most significant M-QTL in individual regressions. An additional two QTL were found in LDHap than in LDmulti (M9, M13). M9 and M13 were the most significant of the remaining M-QTL in individual regressions. M9 had the second highest simulated effect of all the QTL but the lowest MAF and was only identified in LDHap. Hence, in LDmulti and LDHap, power to detect QTL appears to have been mostly limited by effect size.

The QTL identified in LDLA1 show no obvious relationship with MAF, and QTL with both large and small effects were missed (Figure 2). In particular, the two QTL with the largest simulated effects (M9, M15) were not found. In LABayes the same QTL were found as in LDLA1 plus three more (M3, M14, M15). M3 and M15 were found in all the studies except LDLA1. M14 had the lowest simulated effect of the M-QTL and was only detected in LABayes. Eight of the QTL reported in LABayes were also found by LDHap. There were nine QTL found in common between LDBayes and LDHap. LDBayes estimated the position of the first QTL on chromosome 1 closer to M4 than the nearby M5, whereas the estimated location in LABayes was closest to M5. In LDBayes, M11 was also identified, which was not found in any of the other studies.

M7 was not detected in any of the studies. In none of the studies were the two closely linked QTL, M4 and M5, distinguished.

### Accuracy of reported QTL locations

**Figure 3:**
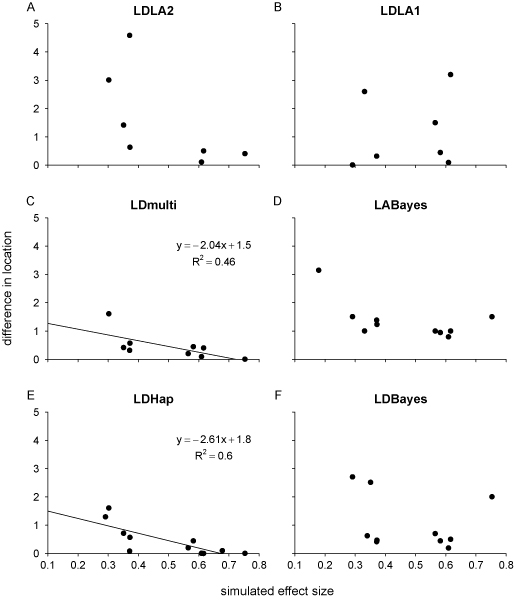
**Accuracy in estimates of QTL location**. In (A)-(F) the absolute difference between the estimated and simulated position for the M-QTL that were detected, is shown in relation to the simulated average effect of allelic substitution, for each study. Lines represent significant relationships (*p *< 0.05 in least squares linear regression) and regression equations and R^2 ^values are given. *P*-values were: (A) 0.12, (B) 0.7, (C) 0.046, (D) 0.07, (E) 0.005, (F) 0.5.

The accuracy in estimates of QTL location was mostly very high (Figure 3). Location estimates in LDmulti and LDHap were most accurate, with the majority of their estimates within 1 cM, and none more than 2 cM, from the correct position. Some of the location estimates in LDBayes, LDLA1 and LDLA2 were very accurate but others were less so. Nearly all the positions reported in LABayes were at least 1 cM from the simulated location, which might be expected as a reduced density of the markers were used. In LDmulti and LDHap, the accuracy of estimated locations increased with the size of the simulated effect.

### Accuracy of reported QTL effects

**Figure 4:**
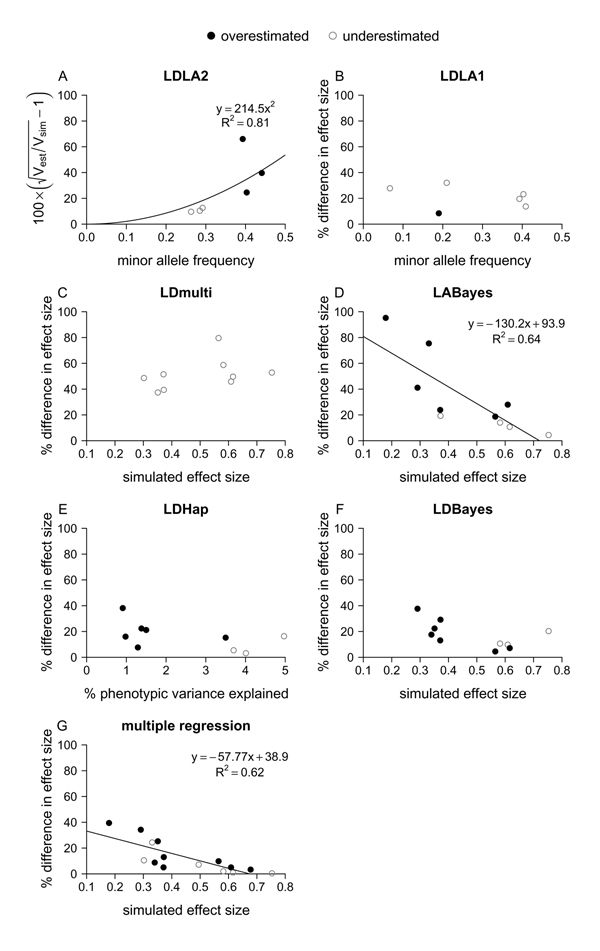
**Accuracy and bias in estimates of QTL effects**. In (A), a function of the square root of the estimated genetic variance of the QTL divided by the simulated genetic variance is shown. In (B)-(G), the difference between the estimated and simulated effect (average effect of allelic substitution) as a percentage of the simulated effect is shown. Potential relationships between the degree of inaccuracy and the simulated effect, minor allele frequency and percentage phenotypic variance explained by the QTL were tested by least squares linear regression for each study and the one with the lowest *p*-value (not dependent on outliers) is illustrated in (A)-(F). In (A), a relationship with the square of minor allele frequency and a zero intercept was fitted. Lines show significant relationships (*p *< 0.05) and regression equations and R^2 ^values are given. Symbols indicate which values were overestimated and which were underestimated. (G) shows the comparable relationship with simulated effect for our multiple regression model. *P*-values were: (A) 0.003, (B) 0.6, (C) 0.3, (D) 0.006, (E) 0.15, (F) 0.07, (G) 0.0005.

Whilst all the studies had good estimates of QTL location for at least some of the M-QTL, there were bigger differences between studies in how well they estimated QTL effects. The most accurate estimates of effect size were in LDLA1, LDHap and LDBayes, which were generally within 30% of the simulated values (Figure 4). They were of comparable accuracy to estimates from our multiple regression for small effect sizes, but slightly less accurate for larger effects.

Effect estimates from LABayes and LDLA2 showed most variation in accuracy. In LABayes, accuracy increased with effect size. Surprisingly, estimates in LDLA2 decreased in accuracy with rising MAF. The overall least accurate estimates of effect size were from LDmulti; they were mostly 40–60% from the actual value.

### Bias in reported QTL effects

In Figure 4, symbols indicate which QTL effects were overestimated and which were underestimated. All the studies showed some indication of bias in effect estimates. In LDmulti, all the QTL effects were underestimated. All but one of the effects were also underestimated in LDLA1. In LDHap and LDBayes (results not shown), QTL effects were overestimated for reported QTL when the variance explained by the QTL was low and underestimated for QTL when the explained variance was high, and there was a similar tendency in LABayes (results not shown). In LDLA2, effects were underestimated for QTL with low MAFs and overestimated for reported QTL with high MAFs. There were no similar signs of any bias in the estimates of effect size from our multiple regression model.

### Epistatic QTL analyses

Searches for epistatic QTL were included in LDmulti and LDHap. A brief summary of the methods used in given in Table 3. Two epistatic pairs were reported in LDmulti and seven in LDHap. No epistasis was simulated in the dataset.

## Discussion

The aim of distributing a common dataset was not to provide an in-depth comparison of alternative methods for QTL analysis, but rather to see how much the results overlapped when different groups performed an exhaustive analysis of a single dataset. Regardless, it is possible to observe marked differences in the power and accuracy of the analyses performed. The results from LDHap were best overall in this dataset. This study had the highest power for a controlled Type 1 error, detected all the M-QTL with the largest effects and had among the best location and effect size estimates. LABayes and LDBayes had the second highest power for QTL detection. However, LDBayes had the highest number of false positives. We reduced the number of putative QTL to include from LDBayes, based simply on effect size, but further work on the reliability of the effects found is needed before this method can be usefully applied in fine-mapping. Despite this, the selected effect size estimates from LDBayes were comparably accurate to those from LDHap and some estimates of QTL location were very accurate, although others were not. LABayes had accurate estimates of large QTL effects but the estimates were much less accurate for small effects. The estimates of QTL location were generally slightly worse than in LDHap and LDBayes, which was probably because only information from every tenth marker was included. The QTL found in LDHap, LABayes and LDBayes were partially complimentary, together accounting for 14 of the 15 M-QTL.

LDHap and LABayes both used information from several markers in detecting QTL, suggesting that such multimarker methods may have higher power to find QTL. However, the results could also be due to specifics of Blossoc and this Bayesian approach. The key gain of the Blossoc method over the other approaches was detection of the QTL at 60 cM on chromosome 3 (M9), which had the second largest simulated effect. Interestingly, this QTL was also reported in LABayes, but it was only found when a lower marker density was used.

LDLA1 had similar accuracy in estimates of QTL effects as LDHap and LDBayes, although fewer QTL were detected. All three studies used single markers as a surrogate for QTL genotype to estimate QTL effects, suggesting that this information is sufficient once QTL have been located. Surprisingly, estimates from the single locus model (LDHap) were as accurate as estimates from the multiple loci models. This may be because a polygenic effect was simultaneously estimated in the single locus model. In our individual regressions on QTL genotype, the estimated effects were mostly less accurate than the estimates from our multiple regression and nearly all overestimated the simulated effect. A multiple locus model can, however, resolve the effect of one QTL from several linked positions. In LDHap, the number of potential QTL was reduced by, arbitrarily, selecting the most significant effect within a 5 cM interval. A single marker analysis was also reported in LDmulti, in which 108 individual markers were significant, but only 9 remained significant in the final multiple regression.

The power of QTL detection was reasonable in LDmulti and estimates of location were accurate. But this study had the least accurate estimates of QTL effects and they were consistently underestimated. Unlike in LDHap and LDLA1, effects were estimated after phenotypes had been pre-corrected for a polygenic effect and this seems to explain the difference. In LDLA1, the estimated variance of the polygenic effect was substantially lower in the final multiple regression model than in the model with no QTL and in LABayes, fitting a polygenic effect as well as multiple QTL gave an estimated polygenic variance close to zero. Both suggest that pre-correcting for the polygenic effect may remove a large part of the variance that could be explained by the QTL. It is recommended that QTL effects estimated by the method in LDmulti, are re-estimated simultaneously with a polygenic effect using the raw data [15]. The impact of pre-correction and its relationship to fitting single and multiple loci should be evaluated in more detail.

LDLA1 and LDLA2, with restricted analyses, had the lowest power. More interesting, however, was which QTL they detected. In both, the aim was described as finding the most important QTL with reduced effort. In actuality, the QTL identified in LDLA1 appeared to be a random sample of the QTL; the QTL with the two largest effects were not detected but several with small effects were. The QTL reported in LDLA2 were those with the highest MAF and several with large effects were missed. In LDLA1 the problem was that for some of the QTL, no nearby markers were selected in the initial screening procedure. The large reduction in marker density also meant that some of the location estimates were poor. In LDLA2 an important limitation was only allowing for two QTL per chromosome. Additionally, only marker data from the last two generations was used and this may explain why some of the location and effect estimates were less accurate in this study. Unfortunately, because of the restrictions imposed in LDLA1 and LDLA2, neither provides a good basis for evaluating LDLA compared to a pure LD or LA analysis.

Aside from LDBayes, the studies had a similar Type I error rate. However, there appeared to be considerable differences in stringency between the approaches. The highest thresholds were applied in LDmulti and LDHap. In LDmulti a Bonferroni correction was used and thresholds corresponding to even lower *p*-values were used in LDHap. It is plausible that more QTL could have been detected in these studies, without an increase in Type 1 error, if lower thresholds had been chosen. It would have been interesting to compare the power and false positive rate of the different analyses with changing thresholds, to see how much of the apparent differences between the methods were a consequence of the threshold choice. Unfortunately, none of the studies gave details of estimates that were judged non-significant. For similar projects in the future, it would be useful to ask participants to provide a list of their top-rated results, irrespective of significance, so that the effect of different thresholds could be investigated. Surprisingly, none of the studies reported permutation analyses to derive chromosome or genome-wide significance levels, although such empirically determined significance thresholds are well established in traditional QTL mapping.

Most studies did not report confidence intervals for estimates of QTL location or effect size. Hence, in assessing which QTL were detected by each study, we imposed a criterion that the estimated location must be within 5 cM of an M-QTL and this limited the amount of location inaccuracy. A preferable method would have been to determine whether a QTL lay within a confidence interval for a given estimate and how the confidence interval for the effect size compared to the simulated effect. This may have been particularly useful in separating estimates with very similar locations. It would also have been interesting to compare the size of the confidence intervals between studies as another means of evaluating the power of the different methods.

One of the aims of the exercise was to explore whether existing methods could distinguish the effects of closely linked loci. Two QTL were simulated at 27.4 and 30 cM on chromosome 2 (M4 and M5, respectively) and they were both detected in our multiple regression model. None of the studies (with the cut-off we applied to LDBayes) reported both these QTL. In LDHap, the minimum distance restriction prevented both from being found and in LABayes there was not enough resolution to separate them. A pair of close, but slightly further apart (6 cM) QTL was reported in LABayes but one of these was a false positive. The reason the two QTL were not detected in LDmulti is probably related to the significance threshold. Developing methods that fully utilise high-density marker maps to correctly identify closely linked QTL remains a future challenge.

Two studies (LDmulti and LDHap) investigated epistasis. All the inferred interactions were false positives. In both studies, tests were performed in two stages, with only the markers passing the first test being tested in the second stage. In LDmulti, the second-round threshold was Bonferroni corrected for the number of pairs passing the first test. In LDHap, an arbitrary threshold of *p *< 10^-6 ^was used. Evidently the thresholds were not sufficiently stringent. Further research is needed on appropriate significance thresholds for epistatic analyses.

## Conclusion

In this dataset, the best methods for detecting QTL were Blossoc [11] followed by a Bayesian linkage analysis [7], both of which used information from multiple markers to infer QTL genotypes. The two studies that aimed to increase the efficiency of QTL detection by reducing the amount of analysis had lowest power and were not effective in identifying the QTL with the largest effects. Estimates of QTL location were generally very good. There were bigger differences in how well the methods estimated the QTL effects. Here, two of the models that were most accurate used single markers in place of QTL genotype and simultaneously fit a polygenic effect. Although in this case estimates from a single locus model were as accurate as from a multilocus model, fitting multiple loci should allow closely linked QTL to be distinguished. A valuable approach might be to first locate QTL by a multimarker/haplotype method and then fit the closest markers in a multilocus model, to estimate QTL effects. For future such projects, we recommend that participants provide a list of their top-ranked effects, and report confidence intervals for QTL location and effect size estimates. Areas that we suggest for further work include significance thresholds, closely linked QTL and epistatic effects.

## List of abbreviations used

GWA: genome-wide association; QTL: quantitative trait loci; LA: linkage analysis; LD: linkage disequilibrium; LDLA: combined linkage disequilibrium and linkage analysis; MAF: minor allele frequency.

## Competing interests

The authors declare that they have no competing interests.
